# The Effect of Body Mass Index on the Degree of Renal Interstitial Fibrosis and Tubular Atrophy - A Retrospective Case-Control Study

**DOI:** 10.7759/cureus.28694

**Published:** 2022-09-02

**Authors:** Reem A Al Zahrani, Faisal K Al Harthi, Faris Irfan Butt, Ahmed D Al Solami, Abdulaziz A Kurdi, Turki O Al Otaibi, Abdulrazaq H Alahmadi, Hanadi Alhozali, Ghada A Ankawi, Mahmoud A Gaddoury

**Affiliations:** 1 Medicine, King Abdulaziz University, Jeddah, SAU; 2 Pathology, King Abdulaziz University Hospital, Jeddah, SAU; 3 Nephrology, King Abdulaziz University Hospital, Jeddah, SAU; 4 Community Medicine, King Abdulaziz University, Jeddah, SAU

**Keywords:** renal health in obesity, obesity and renal tubules, obesity and renal interstitium, bmi and kidney, interstitial fibrosis and tubular atrophy, renal scarring

## Abstract

Introduction

The degree of interstitial fibrosis and tubular atrophy (IFTA) seen on kidney biopsy has long been used to judge the chronicity of kidney disease to predict renal disease outcomes and prognosis. It is an essential component incorporated in many renal disease prognostic classification systems on the native and renal allograft. The impact of increased body mass index on the body metabolism, and the human vascular system, including the functional unit of the kidney, the nephron, is well-addressed in the literature. In this study, we focus on evaluating the degree of IFTA concerning the patient's body mass index (BMI).

Method

All the specimens of nephrectomies performed in King Abdulaziz University Hospital for adults from January 2010 to February 2021 were evaluated for this study. A total of 125 cases were selected for the study. The glass slides were pulled and assessed for the degree of IFTA. The demographic data, and the patient's BMI, were collected from the hospital records.

Results

Subjects with high BMI showed a 1.62 (OR: 1.62, 95% CI: 0.62, 4.22) and 1.52 (AOR: 1.52, 95% CI: 0.56, 4.13) increased risk of high IFTA score compared with those with normal BMI. This study has proved that only at a BMI of 25 or more will there be a measurable, independent effect on the degree of IFTA.

Conclusion

Although a small number of hospital-based populations limits this study, it could prove the increased severity of IFTA in patients with high BMI. Its result may act as a spark that will drive extensive population-based studies that more precisely delineate the relationship between BMI and the degree of IFTA on different levels.

## Introduction

The interstitium supports the blood vessels, renal tubules, and glomeruli. It is composed of a delicate connective tissue matrix. The renal tubules are the urine conducting unit and the site of molecules solutes. Its efficient function is essential for urine production and excretion of solutes [1-2]. Tubular and interstitial injury could be initiated by inflammatory and toxic injury and vascular insufficiency [3]. The outcome of chronic interstitial and tubular injury is the replacement of the interstitium with fibrosis (IF) and tubular atrophy (TA) [4]. The severity of interstitial fibrosis and tubular atrophy (IFTA) measured on kidney biopsy has long been used to judge the chronicity of kidney disease to predict renal disease outcomes and balance the risks and benefits of using immunomodulatory medications [5-7]. It is an item within the Oxford Classification for IgA nephropathy, Banff classification of renal allograft biopsies, and chronicity index of lupus nephritis [5-7]. The direct effect of increased body mass index (BMI) and obesity on renal function has also been studied in different aspects by variable studies in different populations [8-10]. Also, obesity-related glomerulopathy (ORG) is well described in the literature [11]. However, the effect of BMI as an isolated factor on IFTA degree has not been investigated in the literature. This study focused on the relationship between BMI and the degree of IFTA in non-inflammatory kidney specimens. We aim to delineate this relation, describe it, and decide whether the BMI as an independent factor would have a measurable effect on the degree of IFTA and at which BMI figure, the interstitium, and tubules will develop a measurable degree of IFTA. 

## Materials and methods

This study was approved by the Unit of Biomedical Ethics Research Committee in King Abdulaziz University Hospital (KAUH). A case-control study design was used to explore the relationship between the degree of IFTA and the body mass index. We assessed the degree of IFTA on kidney resection cases performed for adult patients only. More than 200 kidney resections were performed in KAUH between January 2010 and February 2021. The cases resected due to renal dysplasia, end-stage renal disease including failed renal graft, and cases with obstructive uropathy, including those with tumors obstructing the pelvis, were excluded from the study. Also, we excluded the cases of partial nephrectomy with less than 5 mm rim of renal parenchyma around the tumor and radical nephrectomy in which the background renal parenchyma was improperly sampled or sampled in close contact with the tumor. We followed these strict selection criteria to avoid the potential pressure effect of the tumor on the renal parenchyma, to avoid obstructive uropathy changes, and to diminish the impact of variability in tumor size on the degree of IFTA. Moreover, cases with a lack of clinical records were also excluded from the study. As a result, we included 125 subjects in the study as cases, and all were resected due to kidney neoplasm.

The data collection process went through two phases. First, the principal investigator, the nephropathologist in the pathology department of KAUH, pulled the hematoxylin and eosin stained glass slides of all the cases from the pathology department store and examined them under light microscopy with careful evaluation of the non-neoplastic kidney parenchyma evaluating the degree of IFTA. 

**Figure 1:**
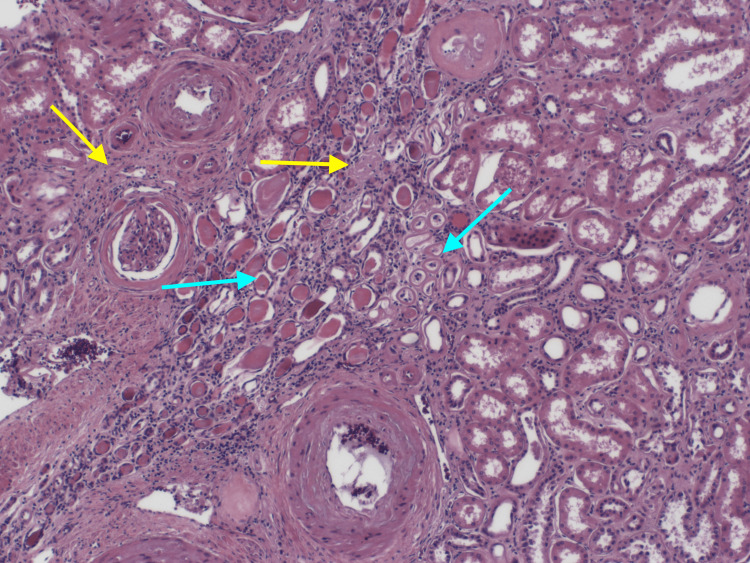
Kidney tissue with an area of interstitial fibrosis and tubular atrophy A light microscopic picture of one of our studied cases, showing kidney tissue with an advanced degree of IFTA and glomerular sclerosis. The yellow arrows indicate the area of interstitial fibrosis, while the blue arrows indicate the atrophic tubules with narrow lumen, hyaline cast, and thick basement membrane.

IFTA definition was adopted from the Banff classification for renal transplant assessment. According to Banff, interstitial fibrosis is the cortex that is replaced by fibrous tissue, while tubular atrophy is defined as tubules with a 50% or more decrease in tubular diameter or tubules with a thickened basement membrane (Figure 1). The degree of IFTA was described as mild, moderate, and severe as classified in the Banff classification [6]; however, due to the small sample size included in the study, the degree of IFTA was used as a binary variable. Cases were defined as those that showed at least 3% IFTA and a control group that showed no element of IFTA at all.

Second, the medical record of the patients was reviewed. Age at the time of the procedure was described as a continuous variable, and gender was described as a binary variable. The patient's clinical information, including the BMI and the systemic comorbidities with well-described impact on the kidney parenchyma, such as diabetes millets (DM), hypertension (HTN), and collagen vascular diseases, were considered during the data collection [12-14]. Only one of the included cases had a collagen vascular disease; rheumatoid arthritis (RA). This case showed 10% IFTA that was not accompanied by any element of interstitial inflammation or glomerulonephritis, which makes it less likely that the IFTA was caused by the pathological effect of RA or its medications. Therefore, only DM and HTN were used in the statistical analysis.

Statistical analysis

Frequencies and percentages (%) were used to describe categorical variables. Mean and standard deviation were used to describe continuous variables. Among the demographic characteristics, age was a continuous variable, while gender was used as a binary variable. In addition, comorbidities, type of procedures, and diagnoses were categorical variables. BMI was used as a binary variable in the analysis due to the small sample size. Those whose BMI was below 25 were considered normal, hence, used as the unexposed group. On the other hand, subjects whose BMI was above 25 were categorized as abnormal (high BMI) and used as the exposed group. 

To examine the association between IFTA score and BMI, bivariate and multivariate logistic regression analysis was conducted using IFTA score as the dependent variable and BMI as the independent variable. Due to their possible confounding effect, age, gender, comorbidities, and type of procedure were included as confounders. The selection of potential confounders for adjustment was based on previous literature and exploring the associations in the data revealed by the directed acyclic (DAGs) graphs [15-16]. The magnitudes of association between the IFTA score and BMI were estimated using odds ratios (ORs) with 95% confidence intervals (CIs). Data analysis was performed using SPSS version 21 (IBM Inc., Armonk, New York).

## Results

Seventy-five (60%) of the cases in this study were male, while 50 (40%) were female. Forty-four (35%) of the patients show some degree of IFTA, ranging from 3% to 50%. Twenty-nine (65%) of cases with IFTA were male, 15 (34%) were female, and 18 (~41%) had no comorbidities. Seventy-four (59%) cases were radical nephrectomies, while 51 (~41%) were partial. Most cases (~61%) were diagnosed as clear cell carcinoma, while the rest were diagnosed with less common tumor types, including oncocytoma, papillary, and chromophobe renal cell carcinoma. Further details are illustrated in Table 1.

**Table 1:** Distribution of demographic characteristics and other variables among subjects with and without IFTA IFTA - interstitial fibrosis and tubular atrophy

Variables	IFTA	
	Yes N (%)	No N (%)
Gender		
Male	29 (38.7%)	46 (61.3%)
Female	15 (30.0%)	35 (70.0%)
Comorbidities		
No	18 (26.9%)	49 (73.1%)
One	12 (41.4%)	17 (58.6%)
> 1	14 (48.3%)	15 (51.7%)
Type of procedure		
Radical	27 (36.5%)	47 (63.5%)
Partial	17 (33.3%)	34 (66.7%)
Diagnosis		
Clear cell carcinoma	28 (36.8%)	48 (63.2%)
Other	16 (32.7%)	33 (67.3%)
Age range: 18-89; age average: 53		

Table 2 shows the risk of high IFTA scores associated with BMI. Both crude and adjusted OR shows a relationship between the exposure and outcome. Without considering confounders, the risk of a high IFTA score associated with BMI was 62% (OR: 1.62, 95% CI: 0.62, 4.22) higher among subjects who scored high BMI compared to those who had low BMI. Also, when measured confounders were controlled for age, gender, type of procedure, and comorbidities, the risk among subjects with high BMI was 52 (OR: 1.52, 95% CI: 0.56, 4.13); higher than those with normal BMI.

**Table 2:** The association between BMI and IFTA *The odd ratio was adjusted for age, gender, type of procedure, and comorbidities IFTA - interstitial fibrosis and tubular atrophy

	IFTA		Crude OR	95% CI	p-value	Adjusted OR*	95% CI	p-value
	Yes (%)	No (%)						
High BMI	37 (37.4%)	62 (62.6%)	1.62	(0.622- 4.220)	0.323	1.527	(0.564-04.137)	0.405
Normal BMI	7 (26.9%)	19 (37.1%)						

Table 3 demonstrates the degree of IFTA concerning the comorbidities. Most of the cases dropped into the mild category of IFTA. In contrast, only one case fell into the category of severe IFTA.

**Table 3:** The degree of IFTA concerning the comorbidities* Most of the cases dropped into the mild category of IFTA. In contrast, only one case fell into the severe IFTA, and few cases exhibited a moderate degree of IFTA. *The total will add up to more than 100% IFTA - interstitial fibrosis and tubular atrophy, DM - diabetes mellitus, HTN - hypertension

Degree of IFTA	DM, N=35	HTN, N=49	DM and HTN, N=24
	N (%)	N (%)	N (%)
3 - <25%	10 (40%)	15 (60%)	6 (24%)
25 - <50%	2 (~67%)	1 (33%)	3 (100%)
50% or more	0	1 (100%)	0

## Discussion

The impact of obesity on the body system health is well described in the literature. It has a well-established rule in developing cardiovascular diseases, metabolic syndromes, diabetes mellitus, and hypertension [17]. The direct effect of obesity on renal function has also been studied in different aspects. A population-based longitudinal cohort study published in 2004 in North America focused on identifying the predictors of chronic kidney disease; 2585 patients with baseline intact kidney function were followed up over 18 months. The body mass index of 27.4 has been proven to increase the odd risk of chronic kidney disease (CKD) onset by 23% (OR, 1.23; 95% CI, 1.08- 1.41) per standard deviation unit [9]. Also, in another North American, more extended cohort study, the participant with higher baseline BMI and those who showed a greater than 10% increase in their body mass index significantly increased the risk of CKD development [10]. A growing body of evidence supporting such a conclusion has accumulated [18]. However, a recent study performed among Asian populations did not show a clear association between high BMI and the risk of chronic kidney disease [19].

Hong et al. have related the higher body mass index to an increase in the degree of mesangial proliferation in Korean patients with IgA nephropathy, which reflect negatively on the MEST-C score and subsequently on the overall patient prognosis [20]. Furthermore, a study conducted in china found that the degree of IF is higher among obese and overweight patients with IgA nephropathy [21]. 

Most of this data negatively relate the high BMI and obesity to the kidney outcome among variable levels and in different disease conditions. The impact of the BMI on the status of the interstitium and tubules needs to be explored. This relationship has not been specifically addressed yet in the literature. Our study focused on elaborating on this relationship, considering the cofounders that may contribute to the BMI effect on renal structure. We could prove that overweight and obese people have an increased risk of developing a severe degree of IFTA. Both crude and adjusted OR have proved this association. The small sample potentially limits this study since it was conducted on cases from one center only, following strict selection criteria. However, this study has undoubtedly added to understanding the impact of high BMI and obesity on kidney substructures and overall function. Furthermore, it plants the seeds for future possibly population-based studies for the more profound elaboration of this relationship to describe the biological basis besides the descriptive one.

## Conclusions

The severity of IFTA seen on kidney biopsy has long been used to judge the chronicity of kidney disease to predict renal outcomes and balance the risks and benefits of using immunomodulatory medications. The impact of obesity on overall renal health is well described. In this study, we emphasized its impact on interstitium and tubules' health status. Relatively the small number of cases and the hospital-based population limits our study. However, this may act as a spark that will drive extensive population-based studies that more precisely delineate this relationship on different levels.
